# Predictive value of serum interleukin-33 and thymic stromal lymphopoietin for the risk of acute exacerbation in patients with chronic obstructive pulmonary disease

**DOI:** 10.3389/fmed.2025.1592734

**Published:** 2025-09-18

**Authors:** Shijia Yin, Kang Xu, Shi Wu, Hua Liu, Zhen Ding

**Affiliations:** ^1^Department of Respiratory and Critical Care Medicine, The Third Affiliated Hospital of Anhui Medical University, Hefei, China; ^2^Anhui Institutes for Food and Drug Control, Hefei, China

**Keywords:** chronic obstructive pulmonary disease, acute exacerbation, interleukin-33, thymic stromal lymphopoietin, risk prediction

## Abstract

**Background:**

Acute exacerbation in patients with chronic obstructive pulmonary disease (COPD) lacks reliable biomarkers. This study aimed to explore the predictive value of serum interleukin-33 (IL-33) and thymic stromal lymphopoietin (TSLP) for acute exacerbations of COPD (AECOPD).

**Methods:**

This retrospective study included patients with stable-phase COPD between June 2023 and June 2024 at the Third Affiliated Hospital of Anhui Medical University. Patients were assigned to the low-risk and high-risk groups according to the number and severity of AECOPD events. Multivariable logistic regression analysis was used to identify the factors of AECOPD risk. Receiver operating characteristic (ROC) curves were used to assess the predictive value of IL-33 and TSLP for AECOPD risk.

**Results:**

A total of 76 patients were enrolled, with 33 in the low-risk group and 43 in the high-risk group. Serum IL-33 (835.30 vs. 770.30, *p* = 0.008) and TSLP (102.20 vs. 90.45, *p* < 0.001) levels were higher in the high-risk group than in the low-risk group. Serum TSLP (odds ratio (OR) = 1.095, 95% confidence interval (CI): 1.013–1.184, *p* = 0.022) was independently associated with AECOPD. The area under the curve (AUC) for IL-33 in predicting AE was 0.677 (95%CI: 0.554–0.801), with 93.0% sensitivity and 39.4% specificity. The AUC for TSLP was 0.768 (95%CI: 0.659–0.878), with 76.7% sensitivity and 72.7% specificity. Combined prediction using IL-33 and TSLP yielded an AUC of 0.779 (95%CI: 0.669–0.888), with 81.4% sensitivity and 72.7% specificity.

**Conclusion:**

High serum TSLP might be associated with an increased risk of AECOPD. While IL-33 alone showed high sensitivity and low specificity, its potential predictive value may be worth exploring.

## Introduction

Chronic obstructive pulmonary disease (COPD) is a common, heterogeneous pulmonary disease characterized by chronic respiratory symptoms due to airway and/or alveolar abnormalities, leading to persistent, usually progressive airflow limitation (1). In 2019, the global prevalence of COPD reached 212.3 million, with over 16 million new cases and more than 3.28 million deaths, making it the leading cause of mortality among chronic respiratory diseases (2). Acute exacerbations (AE) are significant events in the progression of COPD (1). These exacerbations result in a decline in lung function and quality of life and an increase in mortality (3). As the disease advances, both the frequency and severity of acute exacerbations of COPD (AECOPD) rise, contributing to a gradual decline in lung function and severely impacting patients’ quality of life and overall prognosis (1, 3). Treatment for AECOPD can alleviate symptoms but does not prevent lung function deterioration (3). Therefore, early prediction of AE risk and reducing its frequency are key to improving COPD prognosis (4).

Accurate assessment of the condition of COPD patients can help identify and prevent AEs, optimize the allocation of medical resources, reduce the economic burden on patients, and improve their quality of life (5). Available studies suggest that the history of AEs is the most reliable predictor of AECOPD risk (6, 7). However, this approach lacks a pathophysiological explanation of high and low exacerbation risk and does not provide directions for research on clinically targeted therapies (8). Interleukin (IL)-33 is encoded by the IL-33 gene and is a member of the IL-1 family. As an alarmin and a cytokine, IL-33 has a strong ability to drive type 2 inflammation, playing a key role in airway inflammation (9). Type 2 inflammation is involved in many cases of COPD (10). IL-33 mediates immune responses and alveolar damage, and its expression is upregulated in patients with COPD and is associated with disease progression and possibly AE risk (11). Thymic stromal lymphopoietin (TSLP) is a member of the IL-2 family and is released during respiratory epithelial cell damage. It plays a critical role in activating immune cell populations, such as mast cells, eosinophils, and type 2 innate lymphoid cells (12). Elevated TSLP levels have been detected in the serum of patients with COPD and are considered potentially associated with AE risk (12).

Nevertheless, the value of these two biomarkers in predicting AE risk in patients with COPD remains unclear. Therefore, this study aimed to explore the predictive value of serum IL-33 and TSLP for AECOPD.

## Materials and methods

### Study design and patients

This retrospective study included patients with stable-phase COPD treated between June 2023 and June 2024 at the Department of Respiratory and Critical Care Medicine of the Third Affiliated Hospital of Anhui Medical University. The study was approved by the Ethics Committee of the Third Affiliated Hospital of Anhui Medical University (2023–055-01). The requirement for individual informed consent was waived by the committee due to the retrospective nature of the study. The inclusion criteria were (1) diagnosis of COPD based on the Guidelines for Diagnosis and Treatment of Chronic Obstructive Pulmonary Disease (2021 Revised Edition) (13), (2) initially in the stable phase, and (3) clear consciousness and normal communication ability. The exclusion criteria were (1) incomplete clinical data, (2) comorbid asthma, tuberculosis, lung cancer, pulmonary fibrosis, or bronchiectasis, (3) severe dysfunction of the heart, liver, kidneys, or other organs, or (4) mental or cognitive impairments.

Patients were assigned to the low-risk and high-risk groups according to the number and severity of AE. Patients with ≤1 moderate to severe AE (not leading to hospital admission) within 1 year were assigned to the low-risk group, while patients with ≥2 moderate to severe AEs or ≥1 AE that led to hospitalization within 1 year were assigned to the high-risk group (14–17).

### Data collection and definition

Data were collected on sex, age, body mass index (BMI), and the number of hospitalizations due to AE. IL-33 and TSLP levels in the stable phase were determined routinely during the study period using ELISA kits (Guangzhou Huayun Biotechnology Co., Ltd., Guangzhou, China), according to the manufacturer’s instructions. During the study period, pulmonary function was assessed during the stable phase using a MasterScreen spirometer (Jaeger, Germany). The measurements included forced expiratory volume in 1 s (FEV1), FEV1 as a percentage of the predicted value (FEV1%pred), forced vital capacity (FVC), FVC as a percentage of the predicted value (FVC% pred), and the FEV1/FVC ratio. Each patient underwent three repeated tests, and the best result was recorded. The testing procedures adhered to the standards established by the American Thoracic Society (13). All measurements were performed in the context of a routine medical examination for COPD.

The diagnosis of AE was mainly based on the fact that one or more of the main symptoms, such as dyspnea, cough, and expectoration, worsen within a short period (usually several days to weeks), and the degree of change in symptoms exceeds the daily fluctuation range. There are also changes in the characteristics of sputum (such as an increase in sputum volume, purulent sputum, etc.) in COPD patients (18).

### Statistical analysis

Data analysis was performed using SPSS 26.0 (IBM, Armonk, NY, USA). Continuous data with a normal distribution were expressed as means ± standard deviations and analyzed using the independent samples *t*-test. Non-normally distributed data were expressed as medians (interquartile ranges) and analyzed using the Mann–Whitney U-test. Categorical data were expressed as *n* (%) and analyzed using the chi-squared test. Univariable and multivariable logistic regression analyses were performed to identify factors influencing the risk of AECOPD. Variables with *p* < 0.10 in the univariable analyses were included in the multivariable models; the results were presented as odds ratio (OR) and 95% confidence interval (CI). Receiver operating characteristic (ROC) curves were used to evaluate the predictive value of IL-33 and TSLP for AE risk. Two-sided *p*-values <0.05 were considered statistically significant.

## Results

### Characteristics of the low-risk and high-risk groups

**Figure 1:**
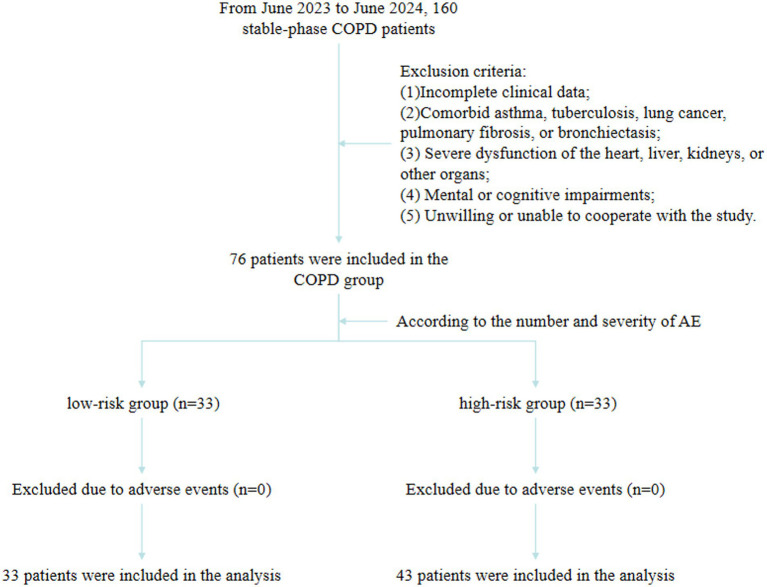
Flowchart for screening research population.

The patients were divided into the low-risk (*n* = 33) and high-risk (*n* = 43) groups based on the occurrence of AE (Figure 1). No statistically significant differences were observed between groups in age, sex, smoking history, family history, white blood cells (WBC), eosinophils (EOS), neutrophil-to-lymphocyte ratio (NLR), C-reactive protein (CRP), or red cell distribution width (RDW) (*p* > 0.05). BMI was significantly higher in the low-risk group compared with the high-risk group (22.96 ± 3.51 vs. 21.09 ± 4.08 kg/m^2^, *p* = 0.039). Serum IL-33 (median, 770.30 vs. 835.30 pg./mL, *p* = 0.008) and TSLP (median, 90.45 vs. 102.20 pg./mL, *p* < 0.001) levels were significantly lower in the low-risk group compared with the high-risk group (Table 1).

**Table 1:** Characteristics of the low risk and high risk groups.

Variable	Low risk	High risk	*p*
Age (years)	75.4 ± 6.76	74.4 ± 6.71	0.505
Sex (*n*, %)			0.962
Male	27 (81.82%)	35 (81.40%)	
Female	6 (18.18%)	8 (18.60%)	
BMI (kg/m^2^)	22.96 ± 3.51	21.09 ± 4.08	0.039
Smoking history			0.676
No-smoking	7(21.21%)	14 (32.56%)	
Mild to moderate smoking history	5 (15.15%)	2 (4.65%)	
Heavy smoking history	21 (63.64%)	27 (62.79%)	
Family history			0.692
Yes	5 (15.15%)	8 (18.60%)	
No	28 (84.85%)	35 (81.40%)	
Pulmonary function			
FVC (L)	1.98 (1.65, 2.33)	2.00 (1.65, 2.25)	1.000
FVC/Pred%	63.80 (52.40, 77.70)	67.40 (55.70, 84.50)	0.593
FEV_1_ (L)	0.92 (0.75, 1.13)	0.89 (0.64, 1.15)	0.332
FEV_1_/Pred%	40.90 (28.80, 63.90)	39.60 (26.10, 57.85)	0.322
FEV_1_/FVC	0.51 (0.39, 0.63)	0.42 (0.37, 0.55)	0.077
WBC (×10^9^/L)	7.77 ± 2.47	7.91 ± 2.64	0.694
EOS (×10^9^/L)	0.08 (0.02, 0.16)	0.09 (0.02, 0.17)	0.983
NLR	3.23 (2.45, 5.26)	4.06 (2.37, 7.41)	0.489
CRP (mg/L)	3.92 (0.50, 12.05)	3.96 (1.25, 12.33)	0.545
RDW (%)	13.27 ± 0.82	13.61 ± 1.21	0.175
IL-33 (pg/mL)	770.30 (678.01, 852.55)	835.30 (756.10, 912.15)	0.008
TSLP (pg/mL)	90.45 (84.88, 96.60)	102.20 (95.50, 110.95)	< 0.001

BMI, body mass index; FVC, forced vital capacity; FEV, forced expiratory volume in 1 s; Pred, predicted; WBC, white blood cells; EOS, eosinophils; NLR, neutrophil-to-lymphocyte ratio; CRP, C-reactive protein; RDW, red cell distribution width; IL-33, interleukin-33; TSLP, thymic stromal lymphopoietin; Mild to moderate smoking history: smoking <20 pack-years; Heavy smoking history: smoking ≥20 pack-years.

*t* for *t*-test, Z for Mann–Whitney U test, and *χ*^2^ for chi-square test.

### Logistic regression analysis of the factors associated with the risk of AECOPD

The univariable logistic regression analyses showed that BMI (OR = 0.879, 95%CI: 0.775–0.998, *p* = 0.039), FEV1/FVC (OR = 0.962, 95%CI: 0.924–1.002, *p* = 0.061), serum IL-33 levels (OR = 1.005, 95%CI: 1.001–1.010, *p* = 0.010), and serum TSLP levels (OR = 1.062, 95%CI: 1.021–1.105, *p* = 0.003) were associated with AECOPD. The variables with *p* < 0.10 in the univariable analyses were included in the multivariable logistic regression analysis. Serum TSLP (OR = 1.095, 95%CI: 1.013–1.184, *p* = 0.022) was identified as independently associated with AECOPD (Table 2).

**Table 2:** Logistic regression analysis of factors associated with the risk of AECOPD.

Variable	Crude OR	95% CI	*p*	Adjusted OR	95% CI	*p*
Age (year)	0.977	0.912–1.046	0.500	–	–	–
Sex						
Male	1.029	0.819–1.239	0.962	–	–	–
Female	Ref	Ref	Ref	–	–	–
BMI (kg/m^2^)	0.879	0.775–0.998	0.039	0.958	0.912–1.006	0.082
Smoking history (years/packs)	1.000	0.999–1.001	0.753			
Family history	0.781	0.230–2.654	0.692			
FVC (L)	0.968	0.391–2.398	0.944			
FVC/Pred%	1.005	0.979–1.032	0.696			
FEV_1_ (L)	0.490	0.142–1.692	0.259			
FEV_1_/Pred%	0.990	0.966–1.013	0.388			
FEV_1_/FVC	0.962	0.924–1.002	0.061	0.887	0.762–1.033	0.123
WBC (×10^9^/L)	1.037	0.867–1.242	0.689			
EOS (×10^9^/L)	2.145	0.127–36.185	0.596			
NLR	1.100	0.971–1.247	0.134			
CRP (mg/L)	0.991	0.936–1.020	0.547			
RDW (%)	1.369	0.867–3.162	0.177			
IL-33 (pg/mL)	1.005	1.001–1.010	0.010	0.996	0.987–1.005	0.433
TSLP (pg/mL)	1.062	1.021–1.105	0.003	1.095	1.013–1.184	0.022

OR, odds ratio; CI, confidence interval; BMI, body mass index; FVC, forced vital capacity; FEV, forced expiratory volume in 1 s; Pred, predicted; WBC, white blood cells; EOS, eosinophils; NLR, neutrophil-to-lymphocyte ratio; CRP, C-reactive protein; RDW, red cell distribution width; IL-33, interleukin-33; TSLP, thymic stromal lymphopoietin.

### Predictive value of serum IL-33 and TSLP for the risk of AECOPD

**Figure 2:**
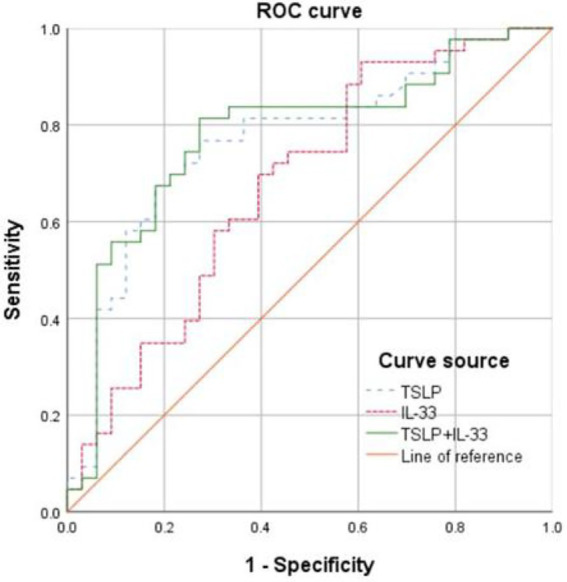
Receiver operating characteristics (ROC) curves showing the predictive value of serum interleukin (IL-33) and thymic stromal lymphopoietin (TSLP) for the risk of AECOPD.

At the optimal cutoff value of 712.95 pg./mL for IL-33, the AUC was 0.677 (95% CI: 0.554–0.801), sensitivity was 93.0%, and specificity was 39.4%. At the optimal cutoff value of 95.33 pg./mL for TSLP, the AUC was 0.768 (95% CI: 0.659–0.878), sensitivity was 76.7%, and specificity was 72.7%. The combined prediction using IL-33 and TSLP yielded an AUC of 0.779 (95% CI: 0.669–0.888), with a sensitivity of 81.4% and specificity of 72.7% (Figure 2 and Table 3).

**Table 3:** Predictive value of serum IL-33 and TSLP for the risk of AECOPD.

Variable	AUC	95%CI	Cutoff	Sensitivity	Specificity
IL-33	0.677	0.554–0.801	712.95 pg./mL	0.930	0.394
TSLP	0.768	0.659–0.878	95.33 pg./mL	0.767	0.727
IL-33 + TSLP	0.779	0.669–0.888	–	0.814	0.727

IL-33, interleukin-33; TSLP, thymic stromal lymphopoietin; AUC, area under the curve.

## Discussion

The results suggested that high serum TSLP is associated with an increased risk of AECOPD. While IL-33 alone showed high sensitivity and low specificity, its potential predictive value may be worth exploring.

The study showed that the average serum IL-33 level in the high-risk group for AE was higher than in the low-risk group. The difference could be associated with the role of IL-33 in the pathogenesis of acute exacerbations in COPD. Indeed, IL-33 is a member of the IL-1 cytokine family and plays a crucial role in innate and adaptive immune responses (9). IL-33 is expressed in endothelial cells, epithelial cells, and fibroblast-like cells, making it present in multiple organs, and its functions in cancer and allergic inflammation are well-recognized (19–21). The epithelial expression of IL-33 is upregulated in asthma and COPD and is associated with disease severity (22). Furthermore, IL-33 promotes inflammation by binding to the IL-33 receptor, growth stimulation expressed gene 2 (ST2), which is present in various cells, including neutrophils, eosinophils, macrophages, basophils, and mast cells. This interaction drives the production of type 2 and non-type 2 inflammatory cytokines, leading to airway smooth muscle contraction, goblet cell mucus production, and fibroblast activation (23), contributing to AEs in COPD. Consequently, the serum IL-33 levels in the high-risk group for AEs were higher than in the low-risk group. Furthermore, the AUC for IL-33 in predicting AE risk was 0.677 (95% CI: 0.554–0.801), with a sensitivity of 93.0% and a specificity of 39.4%. Although the predictive efficiency of serum IL-33 for AE risk is relatively low, its high sensitivity suggests that IL-33 could potentially become a key biomarker and therapeutic target for COPD, pending further confirmation through additional studies. Nevertheless, IL-33 has been identified as a potential therapeutic target for COPD (24–26).

The present study also showed that the serum TSLP levels in the high-risk group for AEs were significantly higher than in the low-risk group. This difference may be related to the pathophysiological role of TSLP in AECOPD. TSLP is produced by epithelial cells in various tissues, such as the lungs, skin, and gastrointestinal tract, as well as by dendritic cells, keratinocytes, stromal cells, basophils, and mast cells (24, 27, 28). TSLP is classified as an “alarmin” and can be secreted by pulmonary structural and immune cells in response to respiratory viruses, air pollutants, allergens, or stimuli such as IL-4, IL-13, and tumor necrosis factor (TNF)-α (29). Elevated levels of TSLP mRNA and protein have been observed in the bronchial mucosa of patients with COPD (30). TSLP has broad immunomodulatory effects, influencing mast cells, type 1 helper T cells, and other cells that produce type 2 and non-type 2 inflammatory cytokines. This process mediates type 2 and non-type 2 inflammation, leading to airway smooth muscle contraction, goblet cell mucus production, and increased excitability of sensory neurons (23), contributing to AECOPD.

This study found that the AUC for TSLP in predicting AE risk was 0.768 (95% CI: 0.659–0.878), with a sensitivity of 76.7% and a specificity of 72.7%. A previous study showed that TSLP could be predictive of AE events but only in patients with low EOS counts. Such a subgroup analysis was not possible in the present study due to the sample size and the retrospective nature of the study. Combining IL-33 and TSLP yielded an AUC of 0.779 (95% CI: 0.669–0.888), with a sensitivity of 81.4% and a specificity of 72.7%, showing limited improvement compared with TSLP alone, suggesting limited added value. Moreover, this study identified serum TSLP as being independently associated with AE, suggesting that TSLP may serve as a novel biomarker for predicting AEs. TSLP (AUC = 0.768) appears to perform similarly to serum amyloid A (AUC = 0.789) and IL-6 (AUC = 0.762) for the prediction of AECOPD progression (31). Some models for predicting AEs in COPD are available [e.g., ACCEPT (31), ACCEPT 2.0 (32), and artificial intelligence (33)], and whether combining them with TSLP and IL-33 levels could improve their predictive value should be investigated. The early identification and timely interventions could reduce the incidence of AE, slow the decline in lung function, improve quality of life, and enhance prognosis. Furthermore, this provides more clinical evidence for targeted therapy in COPD. Nevertheless, additional investigation is necessary since a study showed that high TLSP levels were related to a lower risk of AE in patients with low EOS counts (33).

This study had limitations. It was an observational study with a small sample size. Due to the retrospective nature of the study, the study could only rely on the data available in the charts, and reliable data were missing for too many patients, which could not be used for analysis. Hospitalization in patients with COPD can be used as a surrogate of AE events, as suggested by the literature (14–17), but it is not perfect since the patients can be hospitalized for other causes; nevertheless, in patients with COPD, AE will be the most common cause. While the findings have theoretical significance, larger, multicenter real-world studies are required to validate the roles of IL-33 and TSLP in AECOPD and to further explore their mechanisms. It would provide stronger evidence for the prevention and treatment of COPD.

In conclusion, high serum TSLP levels are associated with an increased risk of AECOPD. The predictive value of IL-33 for AE risk is relatively low, but its high sensitivity suggests potential value. Serum TSLP has a relatively high predictive value. TSLP could be a key biomarker and therapeutic target for AECOPD. Further in-depth, larger-scale, real-world studies are necessary to determine the role of TSLP in controlling AE risk, alleviating lung function decline, improving the quality of life, and prognosis for COPD patients.

## Data Availability

The original contributions presented in the study are included in the article/supplementary material, further inquiries can be directed to the corresponding authors.
